# SCD14-ST and New Generation Inflammatory Biomarkers in the Prediction of COVID-19 Outcome

**DOI:** 10.3390/biom12060826

**Published:** 2022-06-13

**Authors:** Emanuela Galliera, Luca Massaccesi, Lina Yu, Jianwen He, Marco Ranucci, Massimiliano M. Corsi Romanelli

**Affiliations:** 1 Department of Biomedical Sciences for Health, Università degli Studi di Milano, 20133 Milan, Italy; luca.massaccesi@unimi.it (L.M.); mmcorsi@unimi.it (M.M.C.R.); 2 IRCCS Galeazzi Orthopaedic Institute, 20161 Milan, Italy; 3 R&D Department, Mindray Bio-Medical Electronics Co., Ltd., Shenzhen 518057, China; yulina@mindray.com (L.Y.); hejianwen@mindray.com (J.H.); 4 Department of Cardiovascular Anesthesia and Intensive Care, IRCCS Policlinico San Donato, 20097 Milan, Italy; cardioanestesia@virgilio.it; 5 O.U. of Clinical Pathology, Department of Pathology and Laboratory Medicine, IRCCS Policlinico San Donato, 20097 Milan, Italy

**Keywords:** SCD14-ST, SARS-CoV-2, prediction of disease, immune biomarkers

## Abstract

Since no definitive cure for COVID-19 is available so far, one of the challenges against the disease is understanding the clinical features and the laboratory inflammatory markers that can differentiate among different severity grades of the disease. The aim of the present study is a comprehensive and longitudinal evaluation of SCD14-ST and other new inflammatory markers, as well as cytokine storm molecules and current inflammatory parameters, in order to define a panel of biomarkers that could be useful for a better prognostic prediction of COVID-19 mortality. SCD14-ST, as well as the inflammatory markers IL-6, IL-10, SuPAR and sRAGE, were measured in plasma-EDTA of ICU COVID-19 positive patients. In this longitudinal study, SCD14-ST resulted significantly higher in patients who eventually died compared to those who were discharged from the ICU. The results suggest that the new infection biomarker SCD14-ST, in addition to new generation inflammatory biomarkers, such as SuPAR, sRAGE and the cytokines IL-6 and IL-10, can be a useful prognostic tool associated with canonical inflammatory parameters, such as CRP, to predict SARS-CoV-2 outcome in ICU patients.

## 1. Introduction

A novel infectious disease (COVID-19), caused by severe acute respiratory syndrome coronavirus 2 (SARS-CoV-2), was detected in December 2019 and declared a global pandemic by the World Health Organization. Approximately 15% of patients with COVID-19 progress to severe pneumonia and eventually develop acute respiratory distress syndrome (ARDS), septic shock, and/or multiple organ failure with high morbidity and mortality. The COVID-19 pandemic is of significant concern for its extended mortality and for the social and economic effects worldwide. It initially manifests with influenza-like symptoms (fever, dry cough), but it can rapidly worsen into ARDS, which can be lethal [1,2]. Thus far, no definitive cure for COVID-19 is available, and the challenge against the disease is in the early detection, the prompt treatment, and prognostic approach. The innate immune response is the first line of defense against infections [3]. The inflammatory response is mediated by several activated cells of the immune system and regulated by the orchestrated action of pro and anti-inflammatory cytokines, as a self-regulating mechanism, in order to be effective against the pathogen without harming the host [3,4]. An excess of cytokine synthesis leads to an acute severe systemic inflammatory storm, known as a “cytokine storm”, leading to tissue injury. Recent studies have shown that COVID-19 is characterized by a “cytokine storm” syndrome [5,6,7], with an exaggerated release of pro and anti-inflammatory cytokine, resulting in a dysfunction of the immune system and in a multi-organ dysfunction syndrome (MODS) [5,8] as well as several complications of the central nervous system (CNS), including acute encephalopathy [8]. Considering the fast dissemination and the extended mortality, laboratory inflammatory markers that can distinguish severity grades of the disease are needed [9]. In this context, new emerging diagnostic and prognostic markers of infection could be useful in the prediction of the disease severity, as well as pro and anti-inflammatory cytokines involved in the cytokine storm, such as IL-6 and IL-10 [10,11,12]. Among these, an emerging biomarker is SCD14-ST, a soluble CD14 subtype, a marker of sepsis and predictive of disease severity and mortality [13,14]. In addition, a recent study indicates that SCD14-ST can be a useful marker for early diagnosis, risk stratification, and prognosis prediction in pneumonia [15,16]. Only a few recent pieces of evidence suggested a possible role of SCD14-ST as an emerging biomarker in COVID-19 [8,12,16,17]. Only a few recent pieces of evidence suggested a potential role in the definition of COVID-19 severity for other new inflammatory biomarkers, such as SuPAR (soluble urokinase activator receptor), recently defined as a prognostic marker in sepsis and predictive of disease severity in different infections [18,19,20], and sRAGE (soluble receptor for advanced glycation end-products), known as an inflammatory marker with a protective role in several diseases [21,22]. However, no evidence is available so far in the longitudinal evaluation of COVID-19 and mortality. The aim of the present study is a comprehensive and longitudinal evaluation of SCD14-ST and other new inflammatory markers, as well as cytokine storm molecules and current inflammatory parameters, in order to define a panel of biomarkers that could be useful for a better prognostic prediction of COVID-19 mortality.

## 2. Materials and Methods

### 2.1. Study Design and Participants

This longitudinal observational study involved 25 patients affected by COVID-19 admitted to the Intensive Care Unit (ICU) of the IRCCS Policlinico San Donato, in the period March–June 2020. The diagnosis of SARS-CoV-2 infection was confirmed by real-time PCR on the nasopharyngeal swab specimen. The characteristics of the population studied are summarized in Table 1.

Patients were subdivided into two groups according to COVID-19 mortality: 13 patients died before T5 (group A), 12 patients recovered after a longer admission in ICU (group B) and were eventually discharged. The blood draw was performed using evacuated 7-mL plain tubes on all patients (Becton Dickinson, Franklin Lakes, NJ, USA) at T0 (ICU admission) and following the time points ICU T1 (2 days), T2 (7 days), T3 (10 days), T4 (15 days), T5 (20 days) for patients who eventually died. For patients who were eventually discharged, the study could continue to record at further time points until ICU discharge: T6 (30 days), T7 (40 days), T8 (50 days), T9 (60 days), T10 (70 days), T11 (80 days). Plasma + EDTA separation and −80 °C storage were performed on all blood samples.

### 2.2. Quantification of SCD14-ST, IL-6, IL-10, sRAGE, SuPAR, and CRP

SCD14-ST (P-SEP) concentration picogram/milliliter (pg/mL) was measured using CL-1200i (Mindray, Shenzen, China), according to manufacturer protocol based on sandwich immunoenzimatic assay (CLIA). The measurement range of the assay was 20–20,000 pg/mL. IL-6 and IL-10 were measured using an ELISA sandwich assay, according to manufacturer protocols (Bioss antibodies, Boston, MA, USA). sRAGE were measured using an ELISA sandwich Quantikine Assay, according to manufacturer protocol (R&D System, Minneapolis, MN, USA). SuPAR was measured by SuPARnostic ELISA Assay, according to manufacturer protocol (Virogates, Birkeroed, Denmark). CRP was measured using immunoturbidimetric on an automated biochemical analyzer (Olympus CRP-Latex assay, Central Valley, CA, USA).

### 2.3. Statistical Analysis

For all the parameters analyzed, the normality of the distribution of the groups was verified by KS normality. Statistical analysis was performed using a one-way ANOVA test, *p* < 0.05 was considered significant and *p* < 0.005 very significant. Data are expressed as the mean ± standard deviation (SD). Correlation analysis was measured using PRISM 5.0 software by performing linear regression analysis between the different groups of data and calculating the 95% confidence interval of the regression line. The Spearman correlation coefficient (r) was calculated to determine the correlation between values measured by the different assays. Statistical analysis of receiver operating characteristic (ROC) curves and area under the curve (AUC) were performed using PRISM 5.0 software.

## 3. Results

### 3.1. Longitudinal Evaluation of SCD14-ST in COVID-19 Patients

**Figure 1:**
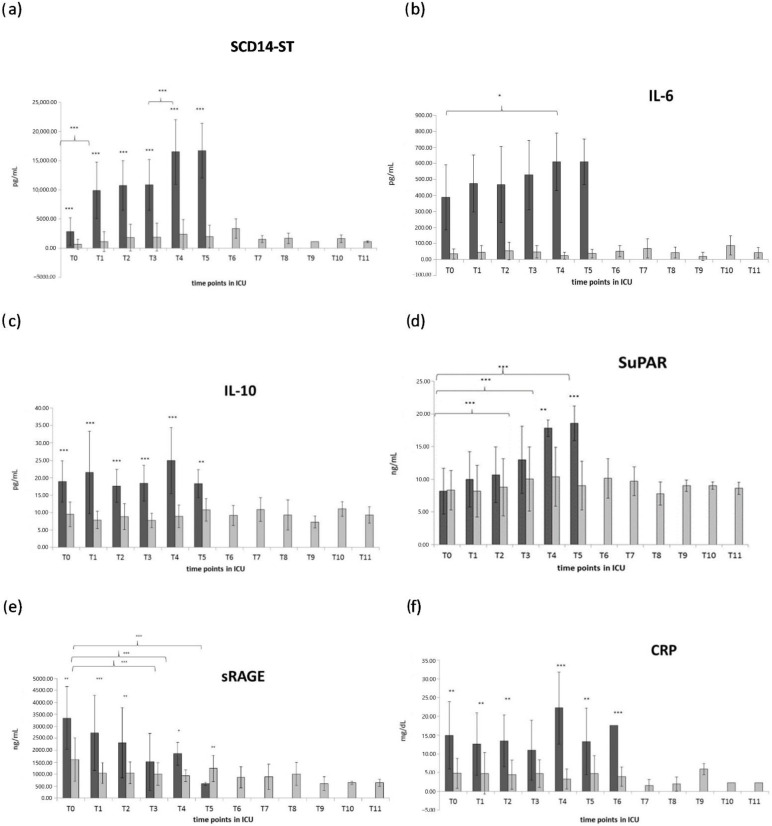
Longitudinal evaluation of sCD14-ST (**a**) and inflammatory markers IL-6 (**b**) IL-10 (**c**) SuPAR (**d**) sRAGE (**e**), CRP: C-reactive protein (**f**), in discharged patients (light gray), and deceased patients (dark grey). (* = *p* < 0.05 quite significative, ** = *p* < 0.01 very significative, *** = *p* < 0.001 extremely significative).

At the moment of admission, SCD14-ST resulted significantly higher in patients who eventually died compared to patients who recovered (*p* < 0.05). In both groups, SCD14-ST values were above the range measured in healthy subjects [23]. At the following time points, SCD14-ST displayed a progressive increase in patients who eventually died (group A); in particular, a statistically significant increase was observed from T0 to T1 (*p* < 0.001) and from T3 to T4 (*p* < 0.001). Conversely, in patients who recovered (group B), SCD14-ST maintained stable levels, similar to T0 levels, all along with the following time points, with no significant variance until the time of discharge from the ICU, as shown in Figure 1a.

### 3.2. Longitudinal Evaluation of Inflammatory Markers in COVID-19 Patients

#### 3.2.1. IL-6

At the time of admission to the ICU, IL-6 showed a strong and significantly higher level in group A compared to group B (*p* < 0.001), as shown in Figure 1b. At the following time points, IL-6 remained stable in group B for all the time points, while it displayed a progressive and gradual increase in group A.

#### 3.2.2. IL-10

At the time of admission to ICU, IL-6 showed a strong and significantly higher level in group A compared to group B (*p* < 0.001), as shown in Figure 1c. At the following time points, IL-6 showed no significant variation in group B. In group A, IL-10 maintained a significantly higher level than group B (*p* < 0.001) at all time points, but it displayed a fluctuating variation without a significant trend of increase along with the time points.

#### 3.2.3. SuPAR

SuPAR showed no significant difference between group A and B in the first three-time points (T0, T1, and T2), while the first significant increase in group A compared to group B appeared at T3. In group A, SuPAR displayed a gradual, though not significant, increase from T0 through the following time points, reaching significantly higher levels T3, T4, and T5 compared to T0, while it remained basically stable, with no significant differences, for all the time points in group B, as shown in Figure 1d.

#### 3.2.4. sRAGE

sRAGE showed a strong and significant difference at time T0 between the two groups, displaying a significantly higher level in group A compared to group B, as shown in Figure 1e. At the following time points T1 and T2, sRAGE displays a gradual and significant decrease, reaching a significantly lower level than group B at T5. In group B, sRAGE displayed a slight but not significantly higher level at T0, compared to the following time points, while from T1 to T11, it showed no significant difference for all of the time points.

#### 3.2.5. C-Reactive Protein

C-reactive protein (CRP) displayed significantly higher levels in group A compared to group B at all time points, as shown in Figure 1e. In group A, CRP showed no significant variation at T1, T2, T3, compared to T0, while it showed a slight but weakly significant increase in T4, and it returned at T5 to levels comparable to T0. In group B, CRP displayed very low levels below the clinical cut-off of 10 mg/dL at all time points, with no significant difference for all the time points.

### 3.3. Severity Score in COVID-19 Patients

Simplified acute pathology scores (SAPS II and SAPS%) were evaluated in the two different groups in order to evaluate the outcome of the deceased and discharged patients and correlate it to the levels of SCD14-ST.

**Figure 2:**
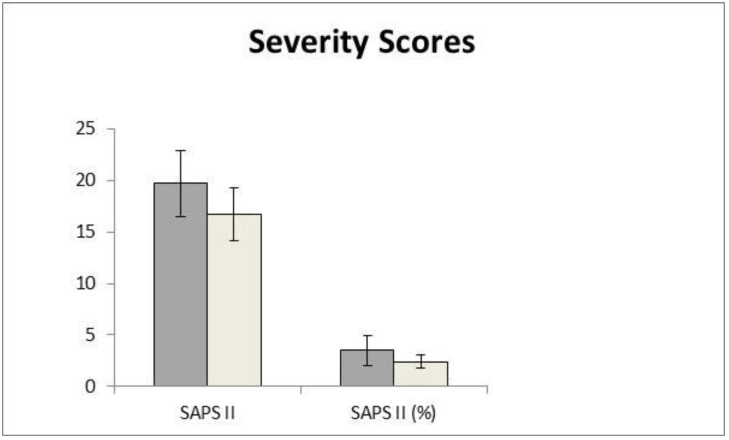
Severity scores SAPS II and SAPS% in the two groups of patients: discharged (light gray), deceased (dark grey).

As shown in Figure 2, the patients who eventually died from COVID-19 disease display a significant increase in both the SAPS II and SAPS% severity scores (19.7 ± 3.2 and 3.5 ± 1.4, respectively) compared to the discharged ones (16.6 ± 2.5 and 2.4 ± 0.6, respectively.

### 3.4. ROC Curve Analysis of Inflammatory SCD14-ST and Inflammatory Markers in COVID-19 Patients

**Figure 3:**
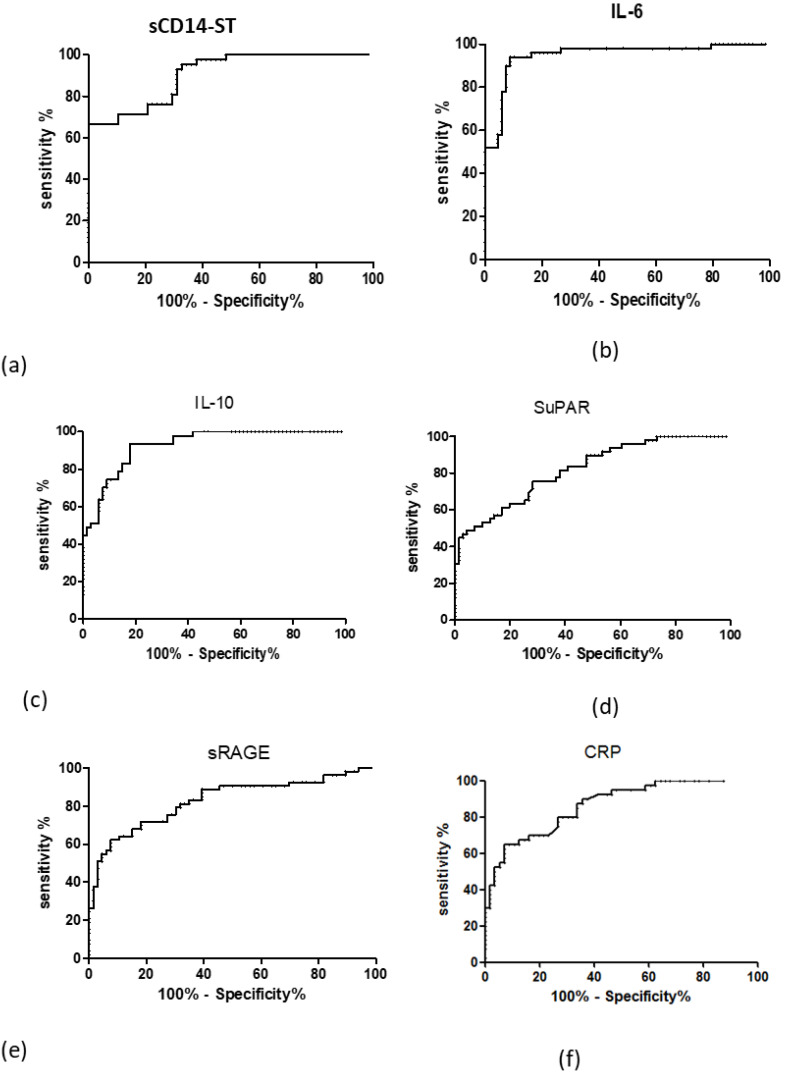
Receiving operating curve (ROC) of SCD14-ST (**a**) and inflammatory biomarkers IL-6 (**b**) IL-10 (**c**) SuPAR (**d**) sRAGE (**e**), CRP: C-reactive protein (**f**).

The diagnostic value and the cut-off of the inflammatory markers have been evaluated by ROC (receiving operative curve) and AUC (area under the curve) analysis, as shown in Table 2. The ROC curves can evaluate the diagnostic efficacy of a diagnostic test by measuring the area under the ROC curve (AUC). In clinical practice, a diagnostic test is considered acceptable if its AUC is ≥0.8 and good if it is ≥0.9. According to this cutoff, as shown in Figure 3, SCD14-ST (panel a), IL-6 ST (panel b), IL-10 (panel c) displayed good AUC (0.906, 0.949, 0.927, respectively), sRAGE (panel e) and CRP (panel e) displayed quite good AUC (0.829 and 0.866 respectively) and SuPAR (panel d) displayed an acceptable AUC = 0.819.

### 3.5. Correlation of SCD14-ST with Inflammatory Markers in COVID-19 Patients

In order to correlate SCD14-ST values with other inflammatory biomarkers, the Pearson correlation coefficient (r^2^) was calculated, and the results are shown in Table 3. sCD14-ST displayed a highly significant positive correlation with CRP and SuPAR (*p* < 0.001), and a significant positive correlation with IL-6 and IL-10 (*p* < 0.01 and *p* < 0.05, respectively), while it displayed a significant negative correlation with sRAGE (*p* < 0.05)

## 4. Discussion

In this study, new potential outcome prediction markers were evaluated in positive COVID-19 ICU patients. The clinical evolution of COVID-19 is still poorly understood, and several aspects of the COVID-19 disease, ranging from mortality to post-COVID-19 impairment, have been recently investigated by different approaches based on biomarkers [8,12].

One of the main challenges in the COVID-19 disease is the prediction of mortality, particularly in hospitalized patients [20]. For this reason, in addition to the current clinical parameters used in patient monitoring, more risk prediction and prognostic factors are needed in order to improve treatment programs for infected patients, in particular those affected by the severe form of the disease who require ICU admission and display the main risk of lethal outcome [21,22].

In the context of markers of infection, an emerging molecule is SCD14-ST, the truncated form of soluble CD14. It was firstly described as a powerful marker of sepsis [23], but its clinical application rapidly extended as diagnostic and prognostic markers for different kinds of infection [11,24]. This molecule is released by macrophages during the inflammatory response to a pathogen, and it correlates with inflammatory cytokine production [25]. For these reasons, this study evaluates whether SCD14-ST could be a good candidate as a mortality risk predictor of COVID-19 disease.

In ICU COVID-19 patients who display a lethal outcome, SCD14-ST displays a significantly higher level at the time of admission in ICU (T0) compared to patients who eventually recovered from the disease, showing good diagnostic potential, as confirmed by the good AUC value. In the longitudinal evaluation, SCD14-ST not only maintained this difference, but it increased over time, reaching the peak at the last time point when the patients eventually died. On the contrary, ICU COVID-19 patients who succeed in recovery from disease maintained a very low level of SCD14-ST all through the longitudinal evaluation until ICU discharge. These results are in agreement with the few recent pieces of evidence describing SCD14-ST in a limited number of patients as a potential prognostic biomarker for COVID-19 pneumonia severity [14,15] and suggest, in addition, the potential role of SCD14-ST as a mortality risk predictor. In agreement with this result, several reports have shown that SCD14-ST is a strong prognostic for the short-term marker of mortality in ARDS [26]. The novelty of this study is the specific application of SCD14ST to ICU COVID-19 patients in a longitudinal evaluation in order to predict the outcome of the disease according to the progressive alteration of serum levels of sCD14ST.

Severity scoring systems are frequently used in intensive care units (ICUs) to assess disease severity, predict mortality, and compare ICU performances [27]. In order to evaluate the outcome of the two groups of patients in the study, severity scores were calculated.

The Simplified Acute Physiology Score II (SAPS II) was developed to help in predicting in-hospital mortality admitted to intensive care units (ICUs) [28]. Severity scores SAPS II and SAPS% were calculated for the patients who eventually died and for the ones who recovered, displaying a statistically significant difference, as shown in Figure 2. SCD14-ST displayed a very good correlation with these severity scores, as shown in Table 3, thus confirming its potential value as a prognostic biomarker for COVID-19 outcomes in ICU patients.

An ideal biomarker of infection should be not only reliable, sensitive, and specific, but it should also be easy and fast in providing a response to a clinical question [29], which is crucial, in particular, for ICU patients. Compared to the other biomarkers evaluated in the study, the advantage of SCD14-ST is that it can be measured by an analytical laboratory instrument very quickly (less than 2 h hours for 100 samples), thus providing a fast response to the ICU clinicians, who can identify COVID-19 patients with a high risk of mortality and adjust the treatment strategy at an early stage. In addition, infection biomarkers are molecules commonly involved in the inflammatory response, and they could lack specificity in distinguishing between infection and inflammation conditions non-related to a specific infection. On the contrary, since the mechanism of secretion of SCD14ST is strictly related to the immune reaction specifically directed against pathogen infection, SCD14ST is very specific for this condition.

Even considering the powerful clinical value of CD14ST in the prediction of mortality of ICU COVID-19 patients, it could be very useful to associate SCD14ST evaluation with a panel of infection and inflammatory biomarkers. It is indeed widely recognized by the literature that a panel of biomarkers in combination is more powerful at defining the clinical condition rather than a single one. [30]. In order to expand the panel of prediction markers of mortality in addition to SCD14-ST, other inflammatory molecules involved in the cytokine storm were evaluated in this longitudinal study. A significant role in the COVID-19 cytokine storm is played by the inflammatory cytokine IL-6 [31], acting as a major player in the systemic effect of pro-inflammatory acute inflammatory response. IL-6 has been extensively studied as an early biomarker of organ dysfunction in sepsis and various acute organ injuries and as a predictive factor of morbidity and mortality in lung diseases [32,33]. IL-6 and IL-10 [34] have been recently described as COVID-19 severity predictors [10]. In this longitudinal study, IL-6 not only displayed good diagnostic power, with a very significant higher level at T0 in patients who eventually died, as confirmed by a high value of the AUC ROC curve but also displayed a gradual increase along with the time points, reaching a stable peak at the last two time points before the patient’s lethal outcome. IL-10 is a pleiotropic cytokine with an immunomodulatory effect, which is produced by a variety of different cell types (macrophages, lymphocytes, fibroblasts) during influenza and sepsis and acute organ injuries [35]. Previous pieces of evidence indicate that IL-10 may be overexpressed in anti-SARS-CoV-2 immunity, being higher in patients with SARS-CoV or MERS, and it could have a prognostic value in predicting disease severity [36]. In this study, IL-10 showed a significantly higher level in patients who eventually died at all time points, but it displayed fluctuations and a gradual increase over time. These results suggest that both IL-6 and IL-10 have a good diagnostic value at T0, as confirmed by their AUC ROC curve, but IL-6 has a better prognostic value in predicting mortality risk compared with IL-10. Previous evidence indicates that IL-10 may be overexpressed in anti-SARS-CoV-2 immunity, being higher in patients with SARS-CoV or MERS [36]

Among prediction makers in infection, another emerging molecule is SuPAR (soluble urokinase plasminogen activator receptor). This molecule is involved in leukocyte recruitment and a coagulation event in the inflammatory response to infection [37]. SuPAR is a soluble molecule that can be easily measured in plasma and serum, reflecting the level of immune system activation [38]. Thus, it is well recognized as a prognostic factor in different kinds of infections, ranging from pneumonia to sepsis [39]. More interestingly, the amount of circulating SuPAR correlates with the severity of the disease, allowing stratification of disease severity [38]. It can predict the elevated risk of acute respiratory distress syndrome ARDS in sepsis, as it correlates with inflammation and mortality [40]. Recent pieces of evidence correlated SuPAR level with COVID-19 pneumonia [1], and it has been suggested as a marker for predicting complications and critical care admission in COVID-19 patients [41]. Consistent with these reports, SuPAR displayed an increasingly higher amount in patients who eventually died compared to ones who recovered. This difference was not evident at the time of admission to the ICU but emerged over time. These results suggest that SuPAR could be considered more a prognostic rather than a diagnostic marker at the moment of admission to ICU, as indicated by the weak AUC ROC curve, and it could be more useful at a later time point to predict the outcome of the COVID-19 disease.

The pathogenetic mechanism of SARS-CoV-2 infection is not fully understood, but several pieces of evidence pointed out the pathogenic role of members of the renin-angiotensin system (RAS) in mediating the susceptibility, infection, inflammatory response, and parenchymal injury in lungs and other organs [42]. The receptor for advanced glycation end-products (RAGE), initially recognized for its ability to bind to advanced glycation end-products (AGEs), is involved in the RAS system and in pathogen-induced pneumonia [43]. RAGE was also recently reported to be directly involved in COVID-19. RAGE is a membrane-bound receptor, and its soluble form, sRAGE, acts as a decoy receptor and disease biomarker [44]. Being a soluble receptor, sRAGE binds AGEs but does not lead to any signaling pathway, thus competing with the signaling, cell-bound RAGE receptor and, as a consequence, limiting the AGEs-RAGE axis detrimental action and tissue damage [45]. For this reason, in many diseases, sRAGE is not only a marker of inflammation but also a protective factor [46]. Being present as a soluble form in circulation, sRAGE can be easily measured and has already been described as a biomarker in several diseases, ranging from cardiovascular to renal and liver disorders and sepsis [47]. In this study, sRAGE showed an initial significantly higher level in patients who eventually died compared to the ones who recovered, confirming the higher inflammatory response in these subjects. These results agree with the previous inflammatory makers evaluated so far in this study. At the following time points, sRAGE showed a significant progressive decrease, therefore reducing its protective role, until the last time point before death, when it displayed a lower level than in patients who recovered, confirming the complete loss of its protective role against organ damages. These results suggest that the decrease in sRAGE from a high level over time could be considered a good prognostic marker for predicting the risk of mortality, as confirmed by the correspondent AUC ROC curve.

In order to compare this new generation biomarker with the current parameter used in the clinical practice, this longitudinal study evaluated C reactive protein as the main inflammatory marker used in the clinical evaluation of patients displaying infections. As shown in Figure 1f, even though CRP displayed significantly higher levels in all of the time points in patients who died, confirming a higher inflammatory response in these patients, it did not show any dramatic increase over the clinical diagnostic cut-off of 10 mg/mL [24,48]. Moreover, CRP did not show a significant increase over time in these patients. These results suggest that CRP alone, even though it can have good diagnostic power at T0, as indicated by the AUC ROC curve, is not alone able to predict a worsening of the disease, but it remains high and stable at each time point until the lethal outcome.

The limitation of the study is the lack of a control group. This is due to the kind of patients selected for the study: they were all recruited from the ICU department, which was entirely dedicated to COVID-19 patients at that time of the first wave of the COVID-19 pandemic.

## 5. Conclusions

Taken together, these results suggest that the new infection biomarker SCD14-ST, in addition to new generation inflammatory biomarkers, such as SuPAR, sRAGE, and the cytokines IL-6 and IL-10, can be a useful prognostic tool associated with canonical inflammatory parameters, such as CRP, to predict the SARS-CoV-2 outcome in ICU patients.

## Figures and Tables

**Table 1:** Clinical and laboratory characteristics of the patient population (n = 25).

Variable	Value	(SD)
Age (years)	63.7	(7.9)
Weight (kgs)	83	(14.3)
Height (cm)	172	(8.3)
Body mass index (kg/m^2^)	28	(4.3)
Baseline creatinine (mg/dL)	1.07	(0.86)
Peak creatinine (mg/dL)	1.7	(1.4)
SAPS II	18	(3.4)
International normalized ratio	1.19	(0.18)
Activated partial thromboplastin time (sec)	36.5	(8.0)
Fibrinogen (mg/dL)	651	(206)
D-Dimer (µg/mL)	4.16	(4.0)
Interleukin 6 (pg/mL)	157	(176)
Platelet count (× 1000 cells/µL)	260	(129)
Ferritin (ng/mL)	1977	(1393)
C-reactive protein (mg/dL)	14.3	(9.5)
Procalcitonin (ng/mL)	3.9	(8.8)
Leukocyte count (cells/µL)	10,539	(5096)
Variable	number of patients	%
Gender male	21	(81%)
Hypertension	11	(42%)
Diabetes	7	(27%)
Chronic obstructive pulmonary disease	5	(19%)
Acute kidney injury	9	(35%)
Obesity	9	(35%)

Data are indicated as mean and standard deviation (SD) or number of patients and % over total number of patients (%), according to parameters characteristics. SAPS: Simplified acute pathology score.

**Table 2:** AUC (area under the curve) of ROC (receiving operating curve) and cut-off of SCD14-ST and the other inflammatory markers evaluated. Correlation (Spearman r, 95% confidence interval) of SCD14-ST with the other inflammatory biomarkers and the severity scores analyzed in the study.

Biomarker	ROC AUC	Cut Off
SCD14-ST	0.906	3853 pg/mL
IL-6	0.946	107.7 pg/mL
IL-10	0.927	12.56 pg/mL
SuPAR	0.829	9.908 ng/mL
CRP	0.866	9.35 ng/dL
sRAGE	0.819	1665 pg/mL

**Table 3:** Correlation (Spearman r, 95% confidence interval) of SCD14-ST with the other inflammatory biomarkers and the severity scores analyzed in the study. (** = *p* < 0.01 very significative, *** = *p* < 0.001 extremely significative).

	IL-6	SuPAR	CRP	IL-10	sRAGE
sCD14ST	0.2715	0.5123	0.3605	0.2207	−0.2291
*p*	0.0321	<0.0001	0.0004	0.00126	0.0146
Significance	**	***	***	***	***

## Data Availability

Not applicable.
